# To adopt is to adapt: the process of implementing the ICF with an acute stroke multidisciplinary team in England

**DOI:** 10.3109/09638288.2012.658489

**Published:** 2012-02-29

**Authors:** Stephanie Tempest, Priscilla Harries, Cherry Kilbride, Lorraine De Souza

**Affiliations:** The Centre for Research in Rehabilitation, School of Health Sciences and Social Care, Brunel University, Uxbridge, United Kingdom

**Keywords:** ICF, health, implementation, stroke

## Abstract

*Purpose:* The success of the International Classifcation of Functioning, Disability and Health (ICF) depends on its uptake in clinical practice. This project aimed to explore ways the ICF could be used with an acute stroke multidisciplinary team and identify key learning from the implementation process. *Method:* Using an action research approach, iterative cycles of observe, plan, act and evaluate were used within three phases: exploratory; innovatory and refective. Thematic analysis was undertaken, using a model of immersion and crystallisation, on data collected via interview and focus groups, e-mail communications, minutes from relevant meetings, feld notes and a refective diary. *Results:* Two overall themes were determined from the data analysis which enabled implementation. There is a need to: (1) adopt the ICF in ways that meet local service needs; and (2) adapt the ICF language and format. *Conclusions:* The empirical fndings demonstrate how to make the ICF classifcation a clinical reality. First, we need to adopt the ICF as a vehicle to implement local service priorities e.g. to structure a multidisciplinary team report, thus enabling ownership of the implementation process. Second, we need to adapt the ICF terminology and format to make it acceptable for use by clinicians.

## Background

The International Classifcation of Functioning, Disability and Health (ICF) [1] has become a globally accepted framework to describe functioning from an integrative biopsycho-social perspective, for example, in rehabilitation [2].

And yet, despite the general endorsement and acknowledgement of the potential use of the ICF in clinical practice [3], there has been no systematic attempt to explain or evaluate the means by which it can be implemented. Furthermore, clinicians need to be convinced of the worth of investing time and fnances into adopting it [4].

**Implications for Rehabilitation**

- The International Classifcation of Functioning, Disability and Health (ICF) is a globally accepted framework to describe functioning and is in use in a variety of clinical settings.
- Yet, the actions necessary to aid the implementation process, with clinicians, have not been explored.
- This study found that an acute stroke multidisciplinary team needed to adapt the ICF and own the way it was introduced within their team, to adopt it into practice.

Training programmes have been established, which are considered an efective way to teach health and social care professionals about the ICF [5,6]. These involve working with an external facilitator with expertise in the ICF. One study concluded that on completion of the training, health care professionals frame their understanding of interventions diferently, with a greater focus on activities and the environment [6]. However, the challenge of understanding the benefts of training is that it remains unclear if the training outcomes, i.e. the greater emphasis on activities and the environment, subsequently transferred into the clinical setting [5]. Moreover, the studies into the efectiveness of training programmes focus on measuring the “before” and “afer” efects, so they provide no insight or guidance for other clinicians wanting to learn about how others have transferred the ICF, as a conceptual framework and classifcation, into clinical practice, or if indeed they have achieved this. Therefore, research is needed to analyse the implementation process.

A number of challenges to adopting the ICF into clinical practice have been discussed in the literature. These refections describe a need for teamwork, a culture change and managerial support [7], as well as a practical requirement to adapt existing artefacts for example assessment documentation [6]. There are problems with the ICF language itself, for example, the negative connotations of the word “functioning” when translated into German [8] and difculties in understanding the ICF terminology for patients with low levels of education or concrete cognitive styles [9]. In addition, it has been suggested that clinicians lack in-depth knowledge and experience in using the framework [4,10]. These insights ofer a hint as to some of the implementation challenges but do not systematically research the learning involved in, or help to inform, the implementation process.

A procedural manual and guide for standardised application of the ICF is being developed to assist practitioners [11], but this process has identifed problematic areas within the ICF; in particular, the overlap of some of the codes and qualifers as well as difculties distinguishing between activities and participation [12]. It could be suggested that one standardised application for the frst edition of the ICF may be too challenging.

As little attention has been directed at understanding how to facilitate the ICF implementation process, the aims of this study were to: (1) identify ways clinicians felt the ICF could be used within their acute stroke multidisciplinary team; and (2) identify the key action processes from trialling it in clinical practice.

## Methods

University Research Ethics Committee and the National Health Service Local Research Ethics Committee granted ethical approval and data was held in accordance with the contemporary data protection legislation.

### Motivations for using action research

An action research approach was deemed to be the appropriate methodological framework for studying the implementation of the ICF with clinicians. Action research has been used in a number of health care studies when evaluating change in practice [13,14] and specifcally in service improvement initiatives for stroke [15–17]. The approach is characterized by three phases: exploratory; innovatory and refective, all of which involve a cyclical process of planning, acting, observing, refecting and re-planning [18]. Furthermore, action research must be participatory and democratic and simultaneously contribute to social science and social change [19].

### Aims

As the project evolved, the following aims were discussed, refned and agreed in negotiation with the research participants:

Exploratory phase: to identify ways the ICF could be used within the acute stroke multidisciplinary team.

Innovatory phase: to develop an ICF-based multidisciplinary report and an ICF glossary.

Refective phase: to evaluate the process of developing these ICF-based clinical tools with an acute stroke multidisci-plinary team.

### Participants

The acute stroke service involved in this research project was established in November 2000. The service included the Stroke Treatment for Every Person (STEP) team; this was an initiative with representation from all the professions involved in local stroke care. The remit of the STEP team is to act as a working party on all service development issues, in line with the recommendations from the National Clinical Guidelines for Stroke [20].

Due to a high level of staf turnover, common in busy, metropolitan teaching hospitals and partly due to rotational posts, only the ward sister, the dietician and the clinical psychologist were in their posts at the start and the end of the project. Existing participants ensured new staf joining the team were informed of the project.

Table I details the participants who chose to engage in the formal data gathering processes for example consenting to interview. Other people chose informal routes of engagement throughout the project for example conversations with the lead researcher, which were subsequently written in the feld notes.

**Table I:** The data collection methods and the participants who chose to engage in the formal data collection procedures.

	Exploratory phase		Innovatory phase	Refective phase
Data collection methods		Documentation analysis (minutes from working party meetings and email communications) Participant based observational field notes Refections from researcher		
	One-to-one semi-structured interviews Focus group employing the nominal group technique			One-to-one semi-structured interviews Focus group employing the force field analysis
Participants	STEP meetings: Representations from all members of the acute stroke multidisciplinary team were present at one or more STEP meetings			
	One-to-one semi-structured interviews (n = 11) comprising: doctor, nurses (n = 2), occupational therapists (n = 2), physiotherapists (n = 2), speech and language therapist, clinical psychologist, carer and family support worker; social worker One focus group (n = 9) comprising: clinical psychologist; psychology assistant; speech and language therapist; dietician; occupational therapists (n = 2); physiotherapists (n = 3)			One focus group (n = 4) comprising: dietician, speech and language therapists (n = 2), physiotherapist One-to-one interviews (n = 3) comprising: clinical psychologist, occupational therapist, dietician (NB: the same dietician from the focus group who wished to expand upon some of the discussions from the focus group)

STEP, Stroke Treatment for Every Person.

Even though the STEP team was the driving force behind this project, they were not the only participants. All members of the wider acute stroke multidisciplinary team were invited to participate and many were involved in diferent ways. In addition, advice and input were sought from a number of people beyond the team including senior managers and information technology specialists. Creating an organisational climate, by engaging with senior managers and key stakeholders, is seen as efective to support and achieve change within stroke care [15].

The frst author (ST) was the lead researcher but, given the democratic nature of action research, was also a participant. A facilitative style of working was adopted, with the frst author drawing upon interpersonal skills, to enable other participants to share their own ideas and views, an approach which has been reported as efective elsewhere in health care action research studies [13]. Previously, as an allied health professional within the stroke team, ST was an insider-outsider researcher i.e. had inside knowledge and experience of working as a therapist within the team but, now employed elsewhere was outside of the daily routine and clinical work.

### Data collection procedure

Table I also outlines the data collection procedure. Topic guides were developed and piloted for the semi-structured interviews and focus groups in the exploratory and refective phases. The exploratory phase interviews asked participants for their opinions on the following topics of interest: written patient-related documentation (i.e. team notes and medical notes); formal patient-related verbal communication (i.e. team meetings and ward rounds); and informal patient-related communication (i.e. *ad hoc* opportunities on the unit such as during joint sessions). These topics were identifed as signif-cant because they focused on communication, the aspect of team work identifed in the National Clinical Guidelines for Stroke [21] where the ICF could be of benefit.

Towards the end of the exploratory phase (a period of approximately 8 months), the nominal group technique was used as the structure for the focus group topic guide, which enabled the evaluation of individual and group strength of opinion [22], thereby ensuring all voices were heard in the process. A single question was posed in the exploratory phase focus group i.e. “In what ways do you think the ICF could be of beneft to the team?”

The refective phase focus group used a diferent topic guide from the exploratory phase; it asked participants for their thoughts on the process of developing the ICF-based tools, and also incorporated a force feld analysis task i.e. asking participants to identify the forces they felt facilitated and hindered the process [23].

**Figure 1:**
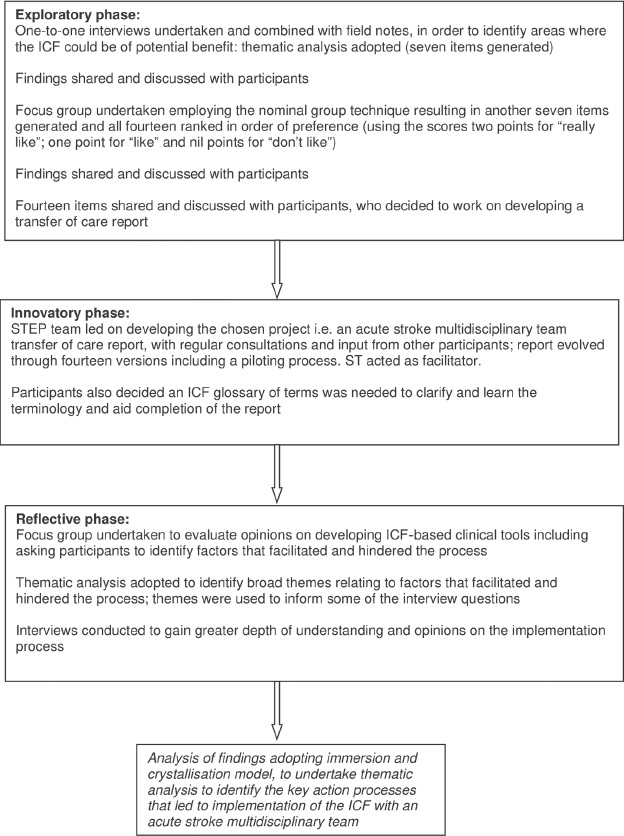
Project procedure. ICF, International Classifcation of Functioning, Disability and Health; STEP, Stroke Treatment for Every Person.

Troughout the feldwork period, feld notes and refections from the researcher were handwritten in A4 notebooks and amounted to fve notebooks of contemporary data entries. Minutes from working party meetings and all emails during this period were stored electronically. Table I outlines the specifc data collection processes undertaken within each phase of the project. Figure 1 outlines the chronological procedure undertaken during the 26-month project.

Consent was obtained for the formal data gathering procedures i.e. the interviews and the focus groups, where a digital voice recorder was used. All data sets were transcribed verbatim. A copy of each transcript was sent to participants, as a form of member checking, to enhance the trustworthiness and transparency of the data collection process [24].

All interviews and both focus groups were conducted at the hospital, in private rooms, at a time identifed as convenient to the participants. The interviews lasted between 30–90 minutes duration and the focus groups were approximately 125 minutes.

### Methods of analysis

Qualitative analysis seeks to provide knowledge and understanding of the phenomenon under study [25] and there are diferent approaches to undertaking it. In this project thematic analysis was the method of choice because, its fexible and pragmatic approach [26] was congruent with the research aims and the nature of action research. In part, this is because thematic analysis has a certain degree of epistemological freedom i.e. it does not rely on an underlying theory such as feminism or neo-Marxism [27].

A conceptual model of “immersion and crystallisation” was adopted; this form of synthesis involves the researcher as a refective participant who is immersed simultaneously in all of the data sets to crystallise overall fndings [28]. In this project, the researcher sought to crystallise the overall theoretical and practical knowledge to evaluate the key action processes and learning points from the development of the ICF-based clinical tools.

In practice, data analysis was undertaken by hand, as the preferred method of the principal researcher when handling a large volume of data. Using the exploratory phase as an example, each data set (e.g. each interview transcript), was read through twice, and initial data were grouped into sub-themes. The initial sub-themes were then refned and grouped in overarching themes which became the frst list of ways the ICF could be used within the service. Units of analysis (i.e. chunks of raw data from various sources) were identifed from the data in relation to each overarching theme. Operational defnitions were used to link each overarching theme and its associated sub-themes to the units of analysis, see example below:

**Table:** 

Overarching theme	Initial subthemes	Operational definition	Sources(from exploratory phase data)
7. To use the ICF as a structure for a transfer of care report	7.1 All writing own reports	There is a joint occupational therapy and physiotherapy report but it is still time consuming and everyone else does their own reports. The nurse has to chase everyone in order to pull them all together before faxing them off and sometimes there is incongruent information on different reports which causes a delay.	Interview 7 pg 4, 13, 14 Interview 9 pg 10 Field notes pgs 2, 38a, 38b, 92, 96, 134

The coding process, operational defnitions, themes and audit trail were shared with experienced researchers to check the transparency of the process, which enhanced the trustworthiness of the data collection process [29]. The fndings were also shared with the participants, who confrmed their authenticity, the STEP team, an audience of experienced researchers within the Centre for Research in Rehabilitation at Brunel University, and members at a local meeting of the Action Research Network.

## Results

Participants identified a number of ways they felt the ICF could benefit their service. Two overall themes were determined as key action processes and change was facilitated by:

1. adopting the ICF in ways that met local service needs.
2. adapting the ICF language and format.

### Adopting the ICF to meet local service needs

One of the key factors in adopting the ICF into practice was for participants to use it to meet their own needs. This involved local ownership of the implementation process, supported by an external facilitator who had experience and knowledge of how to utilise the framework and classifcation. Fourteen ways the ICF could be adopted were identifed by participants and the focus group participants ranked them in order of preference (see Table II).

**Table II:** Ways the ICF could beneft the service as identifed by the exploratory phase participants, in ranked order of preference.

Ways the ICF could be used within an acute stroke multidisciplinary team
To help defne what the service is able to ofer in its acute capacity. To communicate a patient's rehab agenda when referring on. To structure a multidisciplinary team transfer of care report. To identify gaps in the current service provision and target areas for development either within the team or, to see who else can/does ofer a particular service to meet a patient's needs. To guide areas for care planning and goal setting. As a fow chart to guide decision making when referring on. To use in the multidisciplinary team meeting to enhance the structure and provide a written record. As a laminated prompt at the front of the multidisciplinary notes to use as a ready reference. To use as language within the multidisciplinary notes. For a structure in care booklets (as a checklist to see everything is covered) e.g. when a person is being transferred to a nursing home for long term care. To help defne which professionals take the lead in diferent areas of care to assist organising the patient journey. As a “one stop” record of the multidisciplinary team plan. To structure an induction booklet for new staf and students. As a pocket guide for staf to use as a ready reference.

ICF, International Classifcation of Functioning, Disability and Health.

On sharing the ranked list with the wider team, participants subsequently decided on the third way, which was to develop a multidisciplinary team transfer of care report (hereby referred to as “the report”). They hoped that the process of developing the report would also address preferences 1 and 2.

Owning the change process motivated participants to implement the ICF, although the main driver was not to adopt the ICF per se, rather to use it as a vehicle to drive through changes already wanted by the participants i.e. a new report. “*I think the team ownership is really important because, I think it motivates you … if you are allowed to then work with it and try and make it ft with the needs of the team.” (Interview 1 pg. 8)*. Therefore, the ICF was successfully implemented because the participants used it as a subtext to meet their local needs rather than adoption on an explicit level.

It was felt there was a need for an external facilitator to enable successful implementation of the ICF. As the participants were not familiar with the ICF at the start, external support from someone with knowledge and experience of using it helped them to learn the fundamentals. *“It can take a lot of time and energy if you are learning something from scratch, all yourself but then if you are being facilitated by somebody, I mean it's … taken the best bits for our learning and development” (Focus group pg 14 participant 4)*. In addition, it was identifed that an external facilitator avoided the problem with the time and authority required from an internal person taking the lead on facilitating a multidisciplinary project:

Participant 3: “I think it is really hard for an actual therapist to do.” Participant 4: “I mean, the amount of work that has gone into something like this, it is not something that any of us would have the time to do. Tis is such a big thing across all the professions that it would be hard for one speciality to take ownership.” (Focus group discussion pg 26)

### Adapting the ICF: wording and format

Project participants needed to adapt the wording within the ICF and Table III provides an example of the changes made to the wording of the chapter headings within body functions and structures.

**Table III:** Amended headings for the ICF chapters within body functions.

ICF chapter headings for Body Functions	Amended headings on final version of report
Mental functions	Cognition (thinking abilities) Alertness and motivation Mood and behaviour
Sensory functions and pain	Sensory systems and pain
Voice and speech functions	Not included at body level: incorporated into activities and participation. Replaced by: Swallowing
Functions of the cardiovascular, haematological, immunological and respiratory systems	Cardiovascular, haematological, immunological and respiratory systems
Functions of the digestive, metabolic and endocrine systems	Digestive, metabolic and endocrine systems
Genitourinary reproductive functions	Genitourinary/reproductive systems
Neuromusculoskeletal and movement-related functions	Neuromusculoskeletal system and movement
Functions of the skin	Skin condition

ICF, International Classification of Functioning, Disability and Health.

There were two main reasons for changing the ICF terminology: the frst was the need to make it more familiar and user friendly for clinicians, as some of the terms felt separate to the terminology already in use within the service and were not automatically clear. *“It was almost like it was creating a separate language rather than making it easier to understand… these are words we do not use ofen‥‥it was not automatically understandable.” (Focus group pg 4 participant 4)*. Second, some of the terms were changed to make them more acceptable and understandable for patients, their families and carers, as perceived by the participants. Some headings were shortened for example “Functions of the skin” became “skin condition”, the latter being in common use and, as two words, would be easier for a person who had residual communication impairments following their stroke to read and understand. There were also concerns that some of the ICF terminology could be misinterpreted by those receiving the report for example the use of the word “mental” may lead a person to think they had mental health problems in addition to their stroke. *“…things like global mental functions…I think we would perceive that a lot diferently to someone who had had a stroke or family.” (Interview 1 pg 7)*.

The participants also decided that the format of the ICF required adapting to meet their local needs. The ICF chapter headings from body functions, activities and participation, and environmental factors (once adapted by the participants) gave sufcient detail to structure the headings within the report.

The categories within the ICF core set for stroke [30] were considered for use in the report, but even though there were fewer categories than in the ICF full text, participants felt the core set detail would still make the report unwieldy. However, the category level detail within the ICF core set for stroke was not completely abandoned by the participants; through the process of developing the report, they identifed the need to clarify the meaning of each amended ICF chapter heading. Therefore, the categories were subsequently used to develop a glossary, which acted as an aide memoir. The glossary helped the participants to learn the ICF and they felt it could aid consistency when completing the report in the future. *“It was generally felt that some terms* [i.e. adapted chapter headings] *needed further defnition and it would be benefcial to produce a glossary of terms* [i.e. the categories for each chapter heading].”(*Minutes from STEP meeting 16th May 2008*)

**Figure 2:**
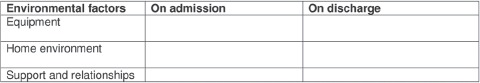
An extract (environmental factors) from the transfer of care report to highlight the use of the (amended) chapter headings.

**Figure 3:**
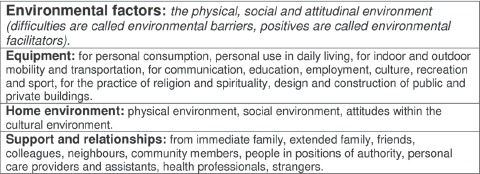
An extract (environmental factors) from the ICF glossary to highlight the use of the ICF core set for stroke, which helped participants to learn the meanings of the chapter headings. ICF, International Classification of Functioning, Disability and Health.

The amended ICF chapter headings became the structure of the transfer of care report, an example, using environmental factors, is shown in Figure 2; the supplementary detail from the glossary is shown in Figure 3.

## Discussion

Our fndings demonstrate a number of key learning points, two of which are particularly signifcant as they bring something new to the debate on ICF implementation: (1) the need to adopt methods of implementation to meet the needs of the local team rather than utilise the core set structure and (2) the need to adapt some of the ICF terminology in the classifcation, to aid clarity and acceptability for clinicians and for patients and their families, as perceived by the clinicians. This project has also provided the frst empirical evidence, to our knowledge, to support the use of an action research approach as a way to facilitate implementation of the ICF into clinical practice.

### Adopting methods of implementation to meet the needs of the local team

At the start of this project, the ICF was not familiar to the participants. In order to identify methods of ICF implementation, the frst challenge is the need for clinicians to learn about the framework and classifcation [4,10]. ICF training programmes have been devised at a national level for example in Italy where over 7000 people have participated in 150 training events [5], but little is known about the direct infuence of these training events on implementing the ICF in clinical practice.

In contrast, this project shows how raising awareness, learning about the ICF and implementation can be successfully combined. Participants learnt about the ICF by focusing on doing i.e. by being involved in a participatory action research process, to provide solutions for practical problems that generated new knowledge. These are key action research principles [19,31]. An action research approach was also an efective method of “convincing” clinicians of the worth of the framework and classifcation, a point of concern which has also been raised within the literature [4].

In the exploratory phase, participants identifed a number of ways that the ICF could be used in their practice. This is in contrast to fndings reported from a psychiatric hospital setting, whereby participants struggled to think of ways to use the ICF [7]. The diferent clinical settings may have impacted on this process, although it is not clear how the information was sought from participants in the latter setting [7]. Furthermore, the National Clinical Guidelines for Stroke [21] recommend the use of the ICF to aid team communication, which may have proved an efective external driver when thinking about why and how to adopt it into acute stroke multidisciplinary practice. Change in practice is likely to be more successful when there is congruence between national and local targets [13].

A number of ways to use the ICF have been highlighted in the literature including its use to “shif from one service to another”, to defne the rehabilitation stages [32], to describe the remit of physiotherapy services [33], to use it as a common language [20] and to structure the cultural artefacts with which multidisciplinary teams identify and protect themselves [34]. These ideas were also identifed by participants in this project who, by prioritising the development of a transfer of care report, identifed the potential importance of using the ICF in moving from one service to another [32], as a common language [20] and as a structure for their cultural artefacts [34].

Previous research has advocated the use of the ICF to communicate the remit of a uniprofessional team [33]. While this project did not seek to do this at the conceptual level, some participants did note the ICF had the potential to outline the remit of not just one profession, but the whole multidisciplinary team.

The ICF has also been advocated to describe the rehabilitation stages [32] and fndings from this project support this idea. Participants reasoned that, by choosing to focus on developing a transfer of care report, it could consequently highlight and outline what their acute service had ofered the person with stroke. At the same time, it could specify and identify subsequent ICF-based areas for future rehabilitation stages. Once the report has been piloted and fully implemented into practice, further research will be required to ascertain whether using the ICF-based tools has led to the outcomes of helping participants to defne team roles and clarify the remit of acute stroke rehabilitation.

More studies are needed that focus on the implementation of the ICF, to inform the most efective ways of adopting it into clinical practice [9]. The importance of publishing evidence on the practical and meaningful applications of the ICF, by those already using it in practice, has also been stressed [4]. The empirical fndings from this project show it was efec-tive to adopt and use the ICF as a subtext or framework to structure previously desired cultural artefacts i.e. a multidisciplinary transfer of care report.

The WHO is developing guidelines and support materials to assist people to implement the ICF [11]. It could be argued that using a “top-down” approach to develop materials [35] may not help people to own the change process, which was key to successful implementation in this research project. However, the WHO is also developing a database for ICF implementation including a section for comments on implementation [11]. If this is an interactive forum whereby learning can occur from sharing experiences of attempts to adopt the ICF, it could provide a way to enhance the ownership of the change process for clinicians.

An extensive discussion on diferent approaches to managing change in the healthcare setting is beyond the scope of this article. However, it has been acknowledged that attention should be paid to change management theory when implementing the ICF [36]. A number of factors that infuence the change process within the healthcare setting have been identifed including the history of the team; the infuence of culture; the threats to roles and the politics of power. The use of processes such as action research can help overcome challenges presented by these factors in order to promote improved and sustained change [34].

### Adapting the ICF

Previous literature has suggested that some of the ICF categories are not easy to understand for patients with low educational levels and concrete cognitive styles [9]; this project adds the concern of understanding for patients with communication difculties following stroke. Our fndings also identifed the need to adapt the terminology for clinicians, thus adding new knowledge to the debate on the user friendliness of the ICF language [8,9].

However, as the project focused on the opinions from one multidisciplinary stroke team, further research is required to ascertain if the ICF language itself is a potential barrier for implementation in clinical practice, or if it does indeed fulfl its original promise of solving the problems caused by professionals using their own technical language [32].

In this project, it was not just the ICF language that needed adapting. The ICF core set for stroke [30] is advocated as the structure for this specifc practice setting having been condensed from the original ICF text to promote clinical utility in stroke [30]. Yet, participants still thought the ICF core set for stroke was too complex and chose to use their locally adapted ICF chapter headings to structure the report. Thus illustrating the core set for stroke did not promote the clinical utility of the ICF for the participants in this study.

The core set format has previously been acknowledged as problematic as it does not have the fexibility to be tailored to individual needs and is time consuming to administer [9]. In this project, a solution was sought whereby the detail of the ICF core set was used as an aide memoir i.e. the glossary to the (adapted) chapter headings. It would be interesting to see if other clinicians, already using the ICF, have experienced similar difculties when using the core set format and to learn how they have sought to overcome any challenges.

In order to adopt the ICF into acute stroke multidisciplinary practice, our fndings demonstrate the need to adapt it. Nonetheless, there have been calls for the WHO to seek proprietary rights for the ICF format and terminology [2], to prevent it being adapted in clinical practice. If this was to happen, the fndings from this project suggest this could present a barrier to adopting the ICF into practice and thus undermine one of the original aims and purposes i.e. to establish a common language in clinical practice.

While the ICF framework was not changed in this project, in adapting the ICF language for local acceptability, it is questionable whether the language remains a common and universal one. The issue of moving away from the ICF language was debated at length by participants. They concluded that any language needs to undergo some form of adaptation process to be used at a local level, just as the English language has a number of regional dialects within the United Kingdom. Furthermore, the Functioning and Disability Reference Group, who advise the WHO on improvements to the classifcation, have proposed work to develop an ICF update platform to gather and process proposals for updates to the ICF [37]; this suggests that information about the need to adapt the ICF could inform these discussions. Indeed, it has been acknowledged that the ICF is an evolving language and on-going dialogue and discussion about its application in practice and the development of its theory is necessary [38]. Therefore, research fndings such as those from this project, could help inform subsequent revisions to enhance the ICF and its uptake in clinical practice.

Finally, there are also philosophical reasons for adapting the ICF in order to encourage a more client-centred approach i.e. to enable people to describe their conditions in their own language [38]. Clinicians, therefore, have a responsibility to adapt frameworks, like the ICF, and clinical tools to facilitate this approach.

### The use of action research

In this project, the use of action research facilitated change, and on a practical level, this meant that local challenges to the implementation process could be defned and overcome. Other methodological frameworks, where the emphasis is not on learning from doing while undertaking action, arguably would not have been able to incorporate practical change management solutions. Rather, learning would be left until completion of the feldwork thus causing a potential time lag when transferring theory into practice.

Nonetheless, the use of action research as a methodological framework has its strengths but also limitations. A strength of this approach lies in the democratic and participatory nature of it, which in this case was a key factor that contributed to the success of the ICF implementation process because participants valued owning the change process. There has been a call for greater collaboration between researchers and clinicians for the development of evidence-based practice, to ensure that research studies have clinical meaning [39] and ground fndings in clinical practice and clinical settings. It was a challenge to engage participation with all professions within the multidisciplinary team as, while all professions were involved in the development of the report to a greater or lesser extent, the hospital management subsequently introduced individual medical and nursing discharge reports, for funding purposes, which meant the fnal report became a therapy transfer of care report. Tis highlights an example of the difculties of implementing change in practice when the local needs of, for example, a stroke service, are incongruent with a wider agenda, i.e. the hospital level requirements [13].

It must also be acknowledged that a limitation of action research is there can be no claim to generalisability, in the traditional sense, with a project that focused on the experiences of a single and established clinical team. As such, individuals must judge the fndings in a diferent way from traditional methods, by considering the relevance and potential impact within the context of their own clinical settings [19].

There were also benefts and shortcomings to having an insider/outsider action researcher. It was advantageous to the research process that, having previously worked in the setting, the researcher understood the historical context of the team and it was arguably easier to build trust to develop new and maintain existing relationships. However, the potential for bias in the analysis of data must not be ignored and to this end, the researcher utilised member checking and the skills of refexivity and self-awareness, both in supervision and with a critical friend, to limit the potential impact.

A further and important challenge to the use of action research is the substantial amount of human resources required in using it to implement the ICF into practice. The duration of the project was 26 months, with completion of the feldwork component in January 2009. However, the transfer of care report and glossary were subsequently piloted then implemented into clinical practice and continued to be in use at the time of writing this article. Therefore, the investment in the process to implement the ICF has brought about sustained change in clinical practice. The use of action research was time efcient because, as previously discussed, this approach enabled participants to learn about the ICF and implement it at the same time. Nonetheless, further research is needed to explore the efectiveness of diferent implementation strategies to adopt the ICF into clinical practice.

## Conclusion

In order to adopt the ICF into clinical practice, participants from an acute stroke multidisciplinary team had to adapt the means of implementation to meet their local service priority needs and adapt the language and format of the ICF. More research is required to (1) explore a range of methods to assist the implementation process to help make the ICF a global clinical reality and (2) to further evaluate the outcomes of implementing the ICF with an acute stroke multidisciplinary team in particular the impact on team work and patient care.
